# Genome Analysis of the Anaerobic Thermohalophilic Bacterium *Halothermothrix orenii*

**DOI:** 10.1371/journal.pone.0004192

**Published:** 2009-01-15

**Authors:** Konstantinos Mavromatis, Natalia Ivanova, Iain Anderson, Athanasios Lykidis, Sean D. Hooper, Hui Sun, Victor Kunin, Alla Lapidus, Philip Hugenholtz, Bharat Patel, Nikos C. Kyrpides

**Affiliations:** 1 DOE- Joint Genome Institute, Walnut Creek, California, United States of America; 2 Microbial Gene Research and Resources Facility, School of Biomolecular and Physical Sciences, Griffith University, Brisbane, Australia; Universidad Miguel Hernandez, Spain

## Abstract

*Halothermothirx orenii* is a strictly anaerobic thermohalophilic bacterium isolated from sediment of a Tunisian salt lake. It belongs to the order Halanaerobiales in the phylum Firmicutes. The complete sequence revealed that the genome consists of one circular chromosome of 2578146 bps encoding 2451 predicted genes. This is the first genome sequence of an organism belonging to the Haloanaerobiales. Features of both Gram positive and Gram negative bacteria were identified with the presence of both a sporulating mechanism typical of Firmicutes and a characteristic Gram negative lipopolysaccharide being the most prominent. Protein sequence analyses and metabolic reconstruction reveal a unique combination of strategies for thermophilic and halophilic adaptation. *H. orenii* can serve as a model organism for the study of the evolution of the Gram negative phenotype as well as the adaptation under thermohalophilic conditions and the development of biotechnological applications under conditions that require high temperatures and high salt concentrations.

## Introduction

Thermohalophiles are organisms that grow optimally at temperatures above 60°C with NaCl concentrations higher than 5%, and are unique amongst extremophiles as they have adapted to this combination of environmental stresses. Study of such organisms will enhance our understanding of survival and adaptation strategies in extreme environments and provide novel biocatalysts with applications in many research, industrial and bioremediation processes [1], [2].

*Halothermothrix orenii* is a Gram negative thermohalophilic, anaerobic bacterium isolated from sediment of a Tunisian salt lake. It grows optimally at 60°C (maximum of 70°C) with 10% NaCl (NaCl growth range between 4–20%). 16S rRNA studies have placed this organism in the order *Haloanaerobiales* [3] in the phylum Firmicutes.

We have determined the complete genome sequence of *H. orenii* and compared it to other organisms with similar phylogenetic and phenotypic features in order to identify the biochemical mechanisms responsible for its adaptation to a hot salty environment. Further, the genome sequence will facilitate the development of *H. orenii* as a model host for cloning and expression of thermohalophilic proteins.

This is the first report of a complete genome for a thermohalophilic bacterium, and for the order *Haloanaerobiales*, which are Firmicutes with a Gram negative phenotype.

## Results and Discussion

### Genome properties

**Figure 1:**
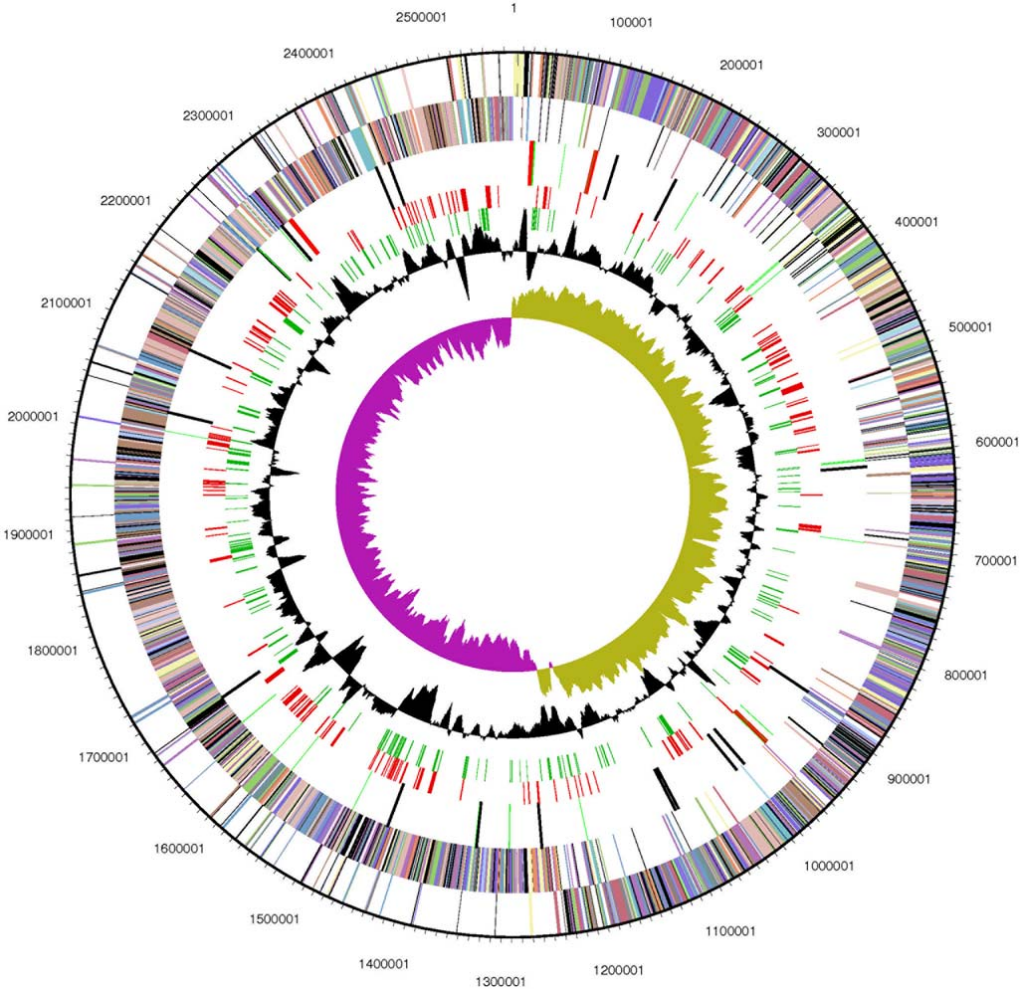
Graphical representation of the chromosome of *H.orenii*. From outside to inside, the first two circles represent the COG assignment for predicted coding sequences on the plus and minus strands, respectively. Colors indicate the following: dark gray, hypothetical proteins; light gray, conserved hypothetical and unknown function; brown, general function prediction; red, replication and repair; green, energy metabolism; blue, carbon and carbohydrate metabolism; cyan, lipid metabolism; magenta, transcription; yellow, translation; orange, amino acid metabolism; pink, metabolism of cofactors and vitamins; light red, purine and pyrimidine metabolism; lavender, signal transduction; sky blue, cellular processes. The third circle represents the RNA genes, red for rRNA and green for tRNA genes. The forth circle corresponds to genes with better Blast hits to genes outside Firmicutes and the fifth circle (red lines) to genes without homologs among the Clostridia. The two innermost circles represent the percent G+C content and G+C skew values, respectively.

The genome consists of one circular chromosome of 2,578,146 bp with a %GC content of 38%. The origin of replication was identified in a region upstream of the DnaA gene where a significant shift in the GC skew value is observed. In the opposite site of the chromosome a similar shift in the GC skew value indicates the presence of the termination of replication site. 2451 genes were predicted, 2366 of which as protein coding genes. 80.6% of genes were assigned to a putative function while the remaining were annotated as hypothetical proteins. The majority of genes are transcribed on the leading strand (2058 - 89%), one of the highest percentages among bacterial genomes (Fig. 1). 311 protein coding genes belong to 122 paralogous families in this genome corresponding to a gene content redundancy of 7.7% a value smaller than the average value for Firmicutes which suggests a more compact organization of the genome in terms of functional content.

There are four rRNA operons with the typical order of 16S, 23S, 5S rRNA genes (Fig. S1). rRNA operon I and IV have identical sequence, while operons II and III are characterized by the insertion of a tRNA coding for isoleucine between the 16S and 23S rRNA genes. Operon III is lacking the 5S rRNA gene suggesting either an incomplete duplication of operon II or a deletion of this site.

Fifty six tRNA genes were identified including cognates for all amino acids. 18 additional RNA genes were predicted using models from the RNA families database (Rfam)[4]. A summary of the properties and statistics of the genome are summarized in Table 1.

**Table 1:** Properties of the *H.orenii* genome.

DNA, total number of bases	2578146	100.00%
DNA coding number of bases	2283859	88.59%
DNA G+C number of bases	976684	37.88%
DNA scaffolds	1	100.00%
Genes total number	2457	100.00%
Protein coding genes	2372	97.24%
RNA genes	85	2.76%
rRNA genes	11	0.45%
5S rRNA	3	0.12%
16S rRNA	4	0.16%
23S rRNA	4	0.16%
tRNA genes	56	2.31%
other RNA genes	18	0.74%
Genes with function prediction	1965	80.57%
Genes without function prediction	407	16,69%
Pseudo Genes	24	0.98%
Fused Genes	105	4.31%
Genes in paralogous clusters	311	12.75%
Genes in COGs	1868	76.59%
Genes in Pfam	1883	77.20%
Genes in TIGRfam	907	37.19%
Genes coding signal peptides	446	18.29%
Genes coding transmembrane proteins	723	29.64%

### Phylogeny

**Figure 2:**
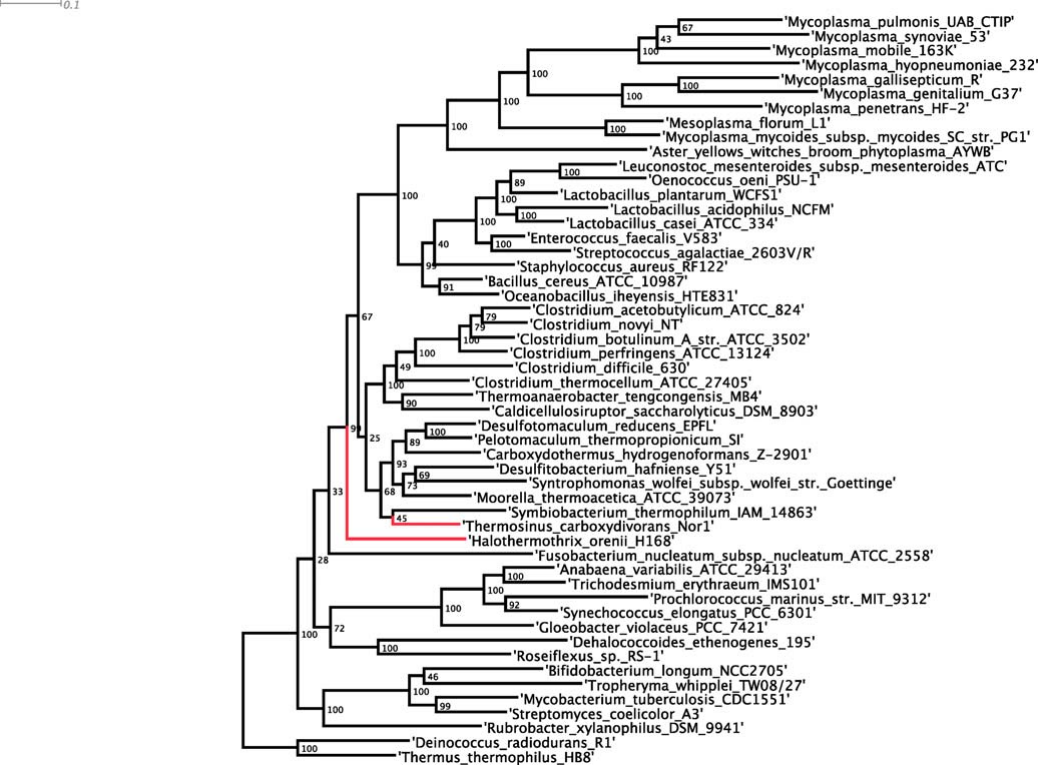
*H.orenii* phylogeny. Maximum likelihood tree of 22 single copy genes from *H.orenii* and 49 reference genomes.

*H. orenii* is currently classified as a member of the order Halanaerobiales in the class Clostridia of the Firmicutes (low G+C Gram positive) phylum on the basis of comparative 16S rRNA sequence analysis [3]. However, the Halanaerobiales is a basal Firmicutes lineage that has been proposed may represent an independent phylum [5]. This suggestion was based on inconsistent support of Firmicutes monophyly when all of its lineages are included in 16S rRNA gene trees either reflecting real polyphyly or the limit of resolution of the single gene (16S) phylogeny. To resolve this question we constructed a maximum likelihood tree of 22 concatenated conserved protein coding genes from *H. orenii* and 49 reference organisms with completed genome sequences. This analysis confirms that *H. orenii* is monophyletic with the other representatives of the Firmicutes (100% bootstrap support), but does not confirm a specific association with the class Clostridia (Fig. 2). Additional representatives of the Halanaerobiales will likely reveal if this order has a specific affiliation to the Clostridia.

### Gram negative features

Of the recognized Firmicutes lineages with Gram-negative representatives Halanaerobiales and Veillonellaceae, only *H.orenii* and *Thermosinus carboxydivorans* have been sequenced respectively. Both contain the pathway for lipid A biosynthesis which is responsible for this phenotype. The genes for lpxA (Hore_17550), lpxB (Hore_17530), lpxC (Hore_17570), lpxD (Hore_17640), lpxK (Hore_18940), and kdtA (Hore_18890) are present. The only reaction in the lipid A biosynthesis scheme without an obvious gene candidate in *H.orenii* is the cleavage of UDP-2,3-diacylglucosamine at its pyrophosphate bond to form 2,3-diacylglucosamine-1-phosphate (lpxH). In contrast to other Gram negative bacteria it contains a unique acyltransferase (Hore_18900) with similarity to lpxL, lpxM and lpxP genes. A lipid A modification system was not identified with the exception of a lipid A 4′-phosphatase (lpxF, Hore_18810) which removes the 4′-phosphate residue from lipid A molecules. A msbA-related protein (Hore_18960) is found in the same operon as the lipid A synthesis enzymes. msbA is required for flipping lipid A from the cytosolic side of the inner membrane to the outer side of the inner membrane.

Phylogenetic trees of orthologs of each of the lipid A pathway genes, as well as of the concatenated alignment of those genes were constructed and compared to the genome tree. In all cases genes from *H.orenii* and *T.carboxydivorans* are monophyletic although the orthologs are not recently diverged (45–65% AA similarity) and genes lpXA, lpXB and lpXC share a unique gene order, including an uncharacterized protein only found in this context in the Firmicutes (Fig. S2). No specific affiliations of the Firmicute lipidA biosynthesis pathway genes to other organisms could be resolved (Fig. S3). This indicates that this pathway in Firmicutes is ancient and highly divergent from the known Gram negative pathways. The most parsimonious interpretation of the common origin and sparse distribution of the lipid A biosynthesis pathway in the Firmicutes (currently only two lineages) is an ancient lateral transfer of the operon between the two groups. A less likely explanation is multiple independent losses of the operon from all other Firmicutes lineages.

Having an outer membrane, *H. orenii* is also unique among Firmicutes in possessing outer membrane secretion proteins of the secretin family. These are the outer membrane subunits of Type II and Type III secretion systems. Two of these secretins (Hore_06090, Hore_18620) appear to belong to large operons containing other type II secretion system proteins. *H. orenii* also has two copies of the chaperone OmpH, a periplasmic protein that helps to transport proteins to the outer membrane.

### Gram positive features

*H.orenii* contains a number of sporulation related genes. Although we were not able to identify homologs of all the genes involved in the sporulation process in Bacilli and Clostridia the main sporulation regulator spo0A is present (Hore_23520) an indication of an active sporulating mechanism. Furthermore, other regulators necessary for the sporulation are present as well as several sporulation related proteins (sporulation stage II, III,IV) and a set of spore germination genes.

It remains an open question if the organism is in fact capable of sporulating under stress conditions, since there is still no experimental evidence to support this. In *C. tetani* E88 the asporogeneous phenotype is attributed to the absence of orphan kinases with transmembrane domains [6]. Thus, although the organism contains the necessary genes for sporulation it is unable to sense the extracellular signals that could trigger the sporulation process. *H.orenii* appears to have five orphan kinases (Hore_00610, Hore_07420, Hore_14600, Hore_19850, Hore_20390) that could play a role in initiating the sporulation cascade.

Some genera belonging to the family Haloanaerobiaceae have also been reported to sporulate (*Sporohalobacter*, *Sporomusa*). We anticipate that the availability of the complete genomic information of *H.orenii* will facilitate research for the elucidation of the underlying mechanisms controlling sporulation coupled to the formation of the Gram negative type outer membrane.

### Adaptation to a hot salty environment

*H.orenii* is the first genome of an anaerobic thermohalophilic organism and its analysis can provide insights into the adaptations of proteins and metabolic pathways to produce this phenotype.

Adaptation to high temperatures is mainly correlated to the production of more stable proteins. This can be achieved by a reduced frequency of the thermolabile amino acids histidine, glutamine and threonine and an increased number of both positively charged and negatively charged residues which suggest that ionic bonds between oppositely charged residues may help to stabilize protein structure at high temperatures [7].

*H. orenii* was isolated from a hypersaline lake, the water of which evaporates during summer and fills during rain leaving microbial life exposed to a fluctuating salinity. Therefore, it can be expected that this moderate halophile has the ability to rapidly adjust to changes in the external salt. In general, two major adaptation strategies to high salinity are observed in halophilic organisms. *Salt-in* strategy involves a shift in amino acid composition, with an increased number of negatively charged residues located on the surface of the enzymes, coupled to the uptake of K^+^ and Cl^−^ and the extrusion of cytotoxic Na^+^ resulting in a cytoplasmic ion composition substantially different from the surroundings. The *salt-out* strategy involves the production of large amounts of specific organic osmolytes (compatible solutes) which can be accumulated to high concentrations without disturbing cellular functions. The *salt-out* strategy is commonly found among organisms that live in environments of moderate salinity or of high salt fluctuations.

The amino acid composition of the proteins in *H.orenii* resembles the profile of thermophilic organisms and is quite distinct from that of the *salt-in* halophilic profiles, suggesting that its proteins have been adapted to high temperatures and to a *salt-out* strategy (Fig. S4). This observation is further supported by genome tag analysis [8] and analysis of protein structures of two thermoactive and halophilic amylases [9]–[11] which revealed that *H. orenii* did not contain high proportions of negatively charged acidic amino acids necessary for a *salt-in* survival strategy. Although this finding was unexpected as the immediate phylogenetic relatives of *H. orenii* [12] are the mesophilic halophiles and hyperhalophiles, all of which contain an excess of acidic charged surface amino acids in their proteins and therefore possess a *salt-in* strategy for survival [8],this type of adaptation to salinity is an indication of the versatility of the organism which allows it to survive in a diverse environment with fluctuations of salt.

Compatible solutes usually can be amino acids and derivatives, small peptides, methylamines, sulfate esters, polyols and sugars. Glycine betaine, proline-ectoine are the most important compatible solutes found in *B. subtilis* and related organisms [13]. Although the metabolic pathways for the production of glycine betaine and ectoine are not present in *H.orenii* a gene coding for a sucrose phosphate synthase (SPS) has been cloned and characterized [14]. Though SPS have also been characterized from photosynthetic cyanobacteria such as Anabaena sp PCC 7120 and *Synechocystis sp* PCC 6803 [15] and presence determined in many other cyanobacterial species, its functional and physiological role in these photosynthetic prokaryotes is speculated to be similar to that for plants, that is, adaptation to osmotic stress. The presence of SPS in bacteria suggests that sucrose synthesis is an ancient trait [15], [16]. It is possible that sucrose also acts as a compatible solute allowing *H.orenii* to maintain an osmotic balance of the cell cytoplasm with the outside environment. *H. orenii* SPS has been selected as a model for investigating evolution and function, and its crystal structure recently solved [17]. Genes encoding for glycine betaine /L proline transport across the membane were also found (Hore_21490 – Hore_21510), suggesting that although *H.orenii* is not able to synthesize these compatible solutes it can acquire them from the environment.

A gene encoding for a protein-L-isoaspartate(D-aspartate) O-methyltransferase (Hore_14460) is also found in *H.orenii* but not in other Firmicutes. This enzyme plays a role in the repair and/or degradation of damaged proteins and can be related to the extremophilic adaptation of this organism. We could not identify pathways for the biosynthesis of thermoprotectants (such as myo-inositol and 2,3-diphosphoglycerate).

Genes encoding for rubrerythrin (Hore_15600, Hore_19080), alkyl hydroperoxide reductase (Hore_18200), two manganese dependent catalases (Hore_23050 and Hore_03960) and a superoxide dismutase (SOD) (Hore_08070) were identified. Rubrerythrin is found in anaerobic sulphate-reducing bacteria and acts as a protectant against oxygen. SOD catalyses the dismutation of superoxide into molecular oxygen and hydrogen peroxide preventing damage from oxygen-mediated free radicals and catalases and alkyl hydroperoxide reductase also exhibit antioxidant activity. Catalases and SOD are involved in antioxidative defense in aerobic and facultative anaerobic microbes but their presence has also been demonstrated in strict anaerobes like *H.orenii*. Strict anaerobes are not uniformly sensitive to oxygen and a broad range of oxygen tolerance exists - some species are very sensitive to oxygen and those at the other end of the spectrum are viable for extended periods in the presence of oxygen. A link between SOD activity and aerotolerance has been noted - the highest SOD activities have been observed in the most aerotolerant strict anaerobe and low SOD activity in those species which are oxygen sensitive [18]. It remains to be determined whether both catalase and SOD are expressed in *H. orenii* and whether the enzyme activities increase in response to dosing the growth medium with oxygen.

### DNA repair

The alpha (polC and dnaE3), beta, gamma/tau, and delta subunits of polymerase III were recognized, this is a typical Gram positive enzyme with implications in the distribution of the genes mainly on the leading strand [19]. Furthermore, DNA polymerase I as well as UmuC (Hore_13250) and UmuD (Hore_11470) homologs, components of DNA polymerase V were also identified.

Other enzymes involved in DNA repair processes such as exodeoxyribonuclease I subunits C and D (Hore_04020, Hore_04030) but not B, RecA and Exonuclease V, UvrB,UvrD(Hore_00850, Hore_04140) as well as DNA IV (Hore_08990) were identified. Excinuclease ABC (Hore_16210 - Hore_16230) is also present while Exonuclease III was not found, although it is common among Firmicutes. A gene for Uracil-DNA glycosylase (Hore_13850) was predicted. Homologs of this gene are found in archaeal genomes and *Clostridium difficile* among the Clostridia. Moreover, a gene coding for a DNA alkylation enzyme was predicted (Hore_01880) which is unique among the Clostridia.

Two genes are predicted to code for photolyases. A photolyase (Hore_04990), responsible for DNA repair at regions that have pyrimidine dimers is not found in other Clostridia and is not common among the Firmicutes. A second photolyase (Hore_22270) has similarity to spore photoproduct lyases, a group of enzymes involved in repair of UV radiation induced DNA damage during spore germination.

### Transcription and translation

The RNA polymerase core enzymes for subunits alpha, beta, beta' and omega and at least eleven sigma factors can be identified. Other transcriptional factors such as the elongation (GreA, Hore_00740), termination (NusA- Hore_07820, NusB- Hore_06300, Rho- Hore_18010) and antitermination (NusG, Hore_01040) factors were also recognized. Using the COG classification more than 70 genes were identified to encode for transcriptional regulators.

Twenty three genes encoding tRNA synthetases for all amino acids were recognized as well as a variety of chaperons such as GroEL/ES, DnaJ/K, GrpE, HslVU, HSP33.

*H. orenii* contains all the major ribosomal proteins. A region of 9.3 Kb, is repeated twice with 100% identity suggesting a recent duplication (Fig. S5). This region harbors two ribosomal proteins (L13 and S9) as well as putative cobalt ABC transporter and a tRNA pseudouridine synthase, a genomic context frequently found among Firmicutes. One of these regions located at position 249693–258969 contains a fragment (pseudogene) of phosphoglucosamine mutase, which is intact in the other region. The sequence identity and the fact that the sequence of the pseudogene has not accumulated any mutations, as one would expect for a non functioning gene suggests a very recently duplication. Detailed analysis of the sequence reads confirmed that this is a real duplication in the genome and not an assembly artifact. These two ribosomal proteins have been identified as single copy genes in all other complete bacterial genomes so far and have also been used as markers for the construction of phylogenetic trees [20]. Accordingly, this is the first time that these two genes are found in more than one copy in a sequenced genome.

### Protein secretion and motility

The Sec AYEG (Hore_16440, Hore_01360, Hore_01030, Hore_15770) system is present. *H.orenii* also possesses genes of a signal-recognition particle (SRP)-like pathway (Hore_07270- Hore_07290) which plays essential role in protein translocation and membrane protein insertion. Two genes of the sec independent twin arginine export pathways, namely TatC (Hore_03800) and TatA/E (Hore_03820) were identified. This corresponds to the minimum requirement for a functional Tat system [21]. Proteins that contain a twin arginine sequence and are predicted to be secreted by the Tat system are listed in table S1 and include a number of proteins related to transport systems and sugar processing (alpha amylases, beta glucosidase) and proteins of unknown functions.

The genome of *H.orenii* contains a complete set of flagellar genes, the majority of which are clustered between the genes Hore_16540 and Hore_17170. The flagellum is powered by the proton pump MotB.

### Central metabolism

*H.orenii* contains all the enzymes necessary for the glycolytic degradation of monosaccharides. Enzymes of the tricarboxylic acid cycle are not present with the exception of a fumarase and no genes of the aerobic respiratory chain are found in common with other anaerobic clostridial genomes. Enzymes for gluconeogenesis and fermentation to ethanol and acetate as well as production of butyrate from branched amino acids are present. Interestingly, a gene coding for a lactate dehydrogenase, exhibiting significant similarity to lactate dehydrogenase genes of *Bacillus* is also found (Hore_16140). However, growth of the organism on lactate has not been observed [3].

A cluster of genes (Hore_14300 – Hore_14350) exhibiting similarity to the Rnf electron transport complex found in other genomes from anaerobic organisms is present. This system is similar to a Na^+^-dependent NADH∶ubiquinone oxidoreductase [22]. Other oxidoreductases include a pyruvate ferredoxin oxidoreductase (Hore_20820), which decarboxylates pyruvate and transfers electrons to ferredoxin molecules, and a NADH dehydrogenase (Hore_14860). This enzyme is a fusion between a NADH dehydrogenase type II [23] and a DoxD domain [24] a membrane spanning domain found in thiosulfate∶quinone oxidoreductases. This enzyme regenerates NAD and transfers electrons to quinone. Electrons can then be used by a hydrogenase to generate H_2_. The genome of *H.orenii* contains the nickel-iron dependent form of hydrogenase, and its two subunits HydA and HydB (Hore_03850, Hore_03860) form a chromosomal cluster with hydrogenase maturation proteins HypA - HypF (Hore_03870, Hore_03920) suggesting a fully functional hydrogenase.

Overall, *H.orenii* can use a large variety of sugar components as energy sources. Genes involved in the metabolism of cellobiose (but not cellulose), starch, glucose, galactose, fructose, fucose, xylose, ribose and citrate were identified. One gene appears to encode a chitinase family 18 protein (Hore_21810) while a second gene exhibits similarity to chitinases (Hore_21670) without having an active site typical for chitinases.

*H.orenii* is capable of *de novo* synthesis of all amino acids except tryptophan. All the enzymes necessary for the biosynthesis of purines and pyrimidines have been identified as well as the enzymes for NAD, coenzyme A and riboflavin biosynthesis.

### Lipid biosynthesis

*H.orenii* uses the non-mevalonate pathway for isoprenoid biosynthesis. All the genes necessary for fatty acid biosynthesis are present as well as copies of the plsX (Hore_10250) and plsY (Hore_10540, Hore_17280) genes for the biosynthesis of phospholipids. A complete set of enzymes for the synthesis of peptidoglycan is also present (mraY, Hore_09060; murF Hore_09050; murC Hore_09100; murD, Hore_09070; murE, Hore_09040; murG, Hore_09090). On the contrary *H.orenii* does not have homologs of the fatty acid beta-oxidation system suggesting that it cannot use fatty acids as carbon sources.

A squalene synthase gene is predicted (Hore_15920). Squalene has been reported to exist in the membranes of archaeal organisms surviving extremely salty environments. Additionally, this gene has similarity to proteins from other halophilic bacteria [25].

### Transporters

*H. orenii* has the highest density of carbohydrate ABC transporters except for members of the Rhizobiaceae, suggesting that sugar metabolism is a primary metabolic process in this organism. Almost 3 percent of the genome encodes carbohydrate ABC transporters. The ABC transporters from family 1 appear to share ATPase subunits as there are 19 sets of binding proteins with permeases, but only 3 ATPases. Only one recent duplication has occurred within these ABC transporters, and almost half appear to have been acquired through horizontal gene transfer, mostly from Proteobacteria. The other transporters either have relatives in Firmicutes, and thus were likely inherited, or do not have close relatives at all.

The phosphotransferase system appears to be in the process of being removed from the genome. There is one IIA subunit and one IIC subunit but no IIB subunit. Enzyme I (Hore_20150) has a frameshift that does not occur at a slippery site, so it is likely to be a pseudogene. Transport by ABC transporters uses more ATP than transport through the PTS system, but ABC transporters have higher affinity. The fact that *H. orenii* is losing the PTS system suggests that it is adapting to low nutrient concentrations.

*H.orenii* has several transporters for amino acids and peptides. It has three members of the sodium∶neurotransmitter symporter family, which in bacteria transport amino acids. One of these is found adjacent to tryptophanase, suggesting that tryptophan can be used for supplemental energy generation. *H.orenii* has two members of the amino acid permease family, one of which is likely to be an arginine/ornithine antiporter. This transporter is found between genes encoding the arginine deiminase pathway, which generates ATP from arginine, with ornithine as a product. Additionally, there is one ABC transporter for amino acids and one for peptides, plus a peptide/proton symporter of the OPT family.

For export of sodium, *H.orenii* has an ABC transporter and a multi-subunit sodium/proton antiporter. A V-type ATPase is also identified. This enzyme is similar to the one found in *Clostridium tetani* [22] which uses ATP to move Na+ cations across the cell membrane. This enzyme in *C.tetani* generates a Na+ motive force at the membrane which is utilized by other transport systems. In *H.orenii* this enzyme could also participate in the desalination of the intracellular space. A member of the NhaC sodium/proton antiporter family was also identified, but this protein is more closely related to a predicted lysine transporter from *Shewanella* (see www.tcdb.org/tcdb/index.php?tc=2.A.35) than to characterized sodium/proton antiporters.

*H.orenii* has the ExbB, ExbD, and putative TonB proteins together on the chromosome. These proteins are involved in energizing transport across the outer membrane in Gram-negative bacteria. While ExbB homologs are found in Firmicutes, and proteins with similarity to part of TonB are also present, *H. orenii* is the only Firmicute to have ExbD. TonB, ExbB, and ExbD interact with outer membrane receptors that transport primarily siderophores, but no receptors of this family could be detected in *H. orenii*, suggesting that TonB/ExbB/ExbD may have a different function in *H. orenii* or that the outer membrane receptors are too divergent to be detected.

*H.orenii* contains 4 copies of the FeoA/FeoB uptake system, an important system for iron supply under anaerobic conditions. In two of the four copies of FeoB the G protein domain exists as a separate protein (Hore_02700, Hore_02710 and Hore_05910, Hore_05920) while in the other two it is fused to the FeoB domain (Hore_13350 and Hore_05070).

### Comparisons to other genomes

We were able to identify 194 genes in *H. orenii* that do not have homologs in any other Firmicutes genomes. The majority of these genes are of unknown function or have a very general functional prediction (table S2). An additional group of 33 *H. orenii* proteins are not found in Clostridia. 196 additional genes have homologs among the Firmicutes but have significantly better hits in genomes outside this phylum. These genes could either reflect the phylogenetic position of this organism as a basal lineage of the Firmicutes or were acquired by horizontal transfer events. Among those are genes related to the Gram negative features of the organism that also exist in *T.carboxydivorans*, sugar transportation and processing enzymes, a sucrose phosphate synthase, a few flagellar genes and DNA repair enzymes. The full list of these genes as well as the phyla with the closest homologs are listed in table S3.

Interestingly, a gene coding for a protein of unknown function (belonging to pfam UPF0236) appears to exist in six identical copies in the genome of H.orenii (Hore_09850, Hore_22880, Hore_12010, Hore_09340, Hore_08430, Hore_07130). Genes similar to these appear also in multiple identical or almost identical copies in other Firmicutes genomes such as species of *Bacillus*, *Geobacillus*, *Lactobacillus*, *Thermoanaerobacter*, *Ammonifex* and *Thermosinus*.

Furthermore, a putative operon consisting of an octopine dehydrogenase (Hore_09740) and a cobalamin B12 binding protein exhibiting distant similarities to methionine synthase (Hore_09750) is exclusively present in *H.orenii* and other thermophilic and anaerobic organisms of the genera *Thermoanaerobacter*, *Petrotoga*, *Roseiflexus*, *Symbiobacterium* and *Thermosipho*. Although the role of those genes is still unknown, their presence in other anaerobic organisms suggests a function related to adaptations to this type of hot anaerobic environments.

### Viruses and CRISPR elements

A CRISPR element was identified at the region 1621710–1623280. In a recent classification of CRISPR sequences to subtypes [26], sequences from *H.oreni* exhibit the highest similarity to sequences of archaeal origin (*Methanosarcina*, *Archaeoglobus*). Next to this repeat region is found a cluster of CRISPR related (cas) proteins with similarity mainly to proteins from thermophilic bacteria and archaea suggesting the relationship of this cas subtype to high temperature environments.

Six proteins exhibit similarity to phage integrases, evidence of phage integration in the genome. (Hore_13740, Hore_00290, Hore_04480, Hore_11080, Hore_03620, Hore_15170). From those genes Hore_13740 is found in a neighborhood with an addiction toxin (Hore_1336a) and a “prevent host death” protein (Hore 13710). Hore_00290 is found next to a transposase IS3/IS911 and an abortive infection protein (Hore_00300); these proteins have been shown to confer resistance to *Lactococcus* phage, in a yet undiscovered mechanism. Finally, Hore _04480 is located next to a transposase and Hore_15170 is found next to the CRISPR region.

There are at least two restriction modification systems on the *H.orenii* chromosome (Hore_04440, Hore_20210) while a third gene fragment of approximately 480 nt (Hore_08500), that is annotated as pseudogene, exhibits distant similarity to a restriction enzyme. Hore_04440 is located next to a transposase, while Hore_20210 is in the same chromosomal neighborhood with plasmid maintenance genes. These observations further support previous suggestions that R-M systems are horizontally acquired and are in constant flux in microbial genomes [27].

### Model host

Halothermophiles can be useful sources of enzymes with biotechnological applications such as sugar processing and hydrolysis after chemical treatment which usually occurs at high temperatures and results in high concentrations of salt. Currently an important limitation, both in research and applications involving thermohalophilic proteins, is the lack of readily available genetic systems for cloning, manipulating and over-expressing such proteins at high temperatures and high salinity. A preferred expression host should not contain restriction modification enzymes that can destroy the foreign DNA, and also have low production of secreted proteases that could hydrolyse the produced proteins. The *H.orenii* genome sequence allows the study and genetic manipulation of such systems in order to increase the yield of cloned products.

*H.orenii* is the first Gram-negative representative of the phylum Firmicutes and the first thermohalophilic organism with a complete genome sequence. The analysis of its genome reveals a mixture of Gram negative and Gram positive properties and provides evidence for the evolution of the Gram negative phenotype. The analysis of its genome provides insights into adaptation strategies at high temperatures and high salinity. Furthermore, it can be used as a model organism for the study of such extremophiles and as a host for the cloning and expression of enzymes under extreme conditions.

## Materials and Methods

### Culture and DNA extraction

*Halothermothrix orenii* was grown under strict anaerobic conditions and DNA extracted and purified using a modification of the method of Marmur as previously described [8], [9].

### Genome sequencing, assembly and annotation

The genome of H.orenii was sequenced at the Joint Genome Institute (JGI) using a combination of 3-kb and fosmid (40-kb) libraries and 454 FLX pyrosequencing All general aspects of library construction and sequencing performed at the JGI can be found at http://www.jgi.doe.gov/. The Phred/Phrap/Consed software package (www.phrap.com) was used to assemble 3-kb and fosmid libraries and to assess quality (Ewing and Green 1998; Ewing et al. 1998; Gordon et al. 1998). Possible mis-assemblies were corrected, gaps between contigs were closed by custom primer walks from sub-clones or PCR products. The error rate of the completed genome sequence of *H.orenii* is less than 1 in 50,000.

Genome annotation was performed with the Integrated Microbial Genomes Expert Review (IMG-ER) annotation pipeline (http://img.jgi.doe.gov/w/doc/img_er_ann.pdf). Predicted coding sequences (CDSs) were additionally manually evaluated using JGI's Quality Assurance pipeline (http://genomebiology.jgi-psf.org/Content/QApipeline.htm).

#### Genome analysis

Comparative analyses of *H.orenii* with related organisms was performed using tools and reference genomes available in the Integrated Microbial Genomes system (IMG) v.2.2 [28]. Unique and homologous *H.orenii* genes were identified by using BLASTp (cutoff scores of E<10^−2^). Reciprocal hits were calculated based on these values. Comparisons between genes for the identification of common genes were done using BLAST similarities of e-value 10^−5^ and similarity >20%. Signal peptides were identified using the SignalP 3.0 [29] and TMHMM [30] at default values.

Habitat information for sequenced organisms was retrieved from the GOLD database [31] to correlate with gene content. Comparison of residue frequencies was done using a chi square test.

Genome redundancy was calculated as the percentage of redundant genes in a genome [Gr = (genes in paralogous groups – number of paralogous groups)/total gene content ].

A “whole genome” tree was based on a previously published list of 31 universally distributed, single-copy genes, that are resistant to horizontal transfer [20]. Twenty-two of these genes were concatenated, and aligned by the program MUSCLE [32]. Masks were created in ARB [33] using the base frequency filter tool (30% minimal similarity) to remove regions of ambiguous positional homology from all amino acid alignments. This produced a masked alignment of 3652 amino acids for phylogenetic inference. Maximum likelihood trees were created using TREE-PUZZLE [34] and RaxML [35]. For RAxML trees, 100 bootstrap resamplings were used to estimate confidence of the inferred topology.

Phylogenetic trees of genes of the lipid A biosynthesis pathway were performed between the best reciprocal hits of genes to the *H.orenii* counterparts in all the genomes that contained all genes of the lipidA biosynthesis pathway. Alignments were performed using the program MUSCLE and Maximum likelihood phylogenetic trees calculated using ARB using the SAI filter described previously.

#### Nucleotide sequence accession numbers

The sequence data described here have been deposited in GenBank (CP001098) and the Genomes On Line Database accession number Gc01049.

## Supporting Information

Figure S1 The ribosomal operons in *H.orenii*. Red: 16S RNA, Blue: 23S RNA, Green: 5S RNA.
(0.03 MB DOC)

Figure S2 Gene order around genes belonging to the lipidA pathway. Genes for *H.orenii* have been marked. Homologous genes in other genomes are indicated with same colors.
(0.04 MB DOC)

Figure S3 Neighbor-joining tree of concatenated sequences of genes belonging to the lipidA pathway.
(0.23 MB DOC)

Figure S4 Residue frequency in Mesophilic, Thermophilic, and halophilic bacteria compared to residue frequency in *H.orenii*. White boxes indicate residues with significant difference in their abundance between the different classes of organisms.
(0.15 MB DOC)

Figure S5 Recent duplication in *H.orenii* genome. i. cobalt transporter, ii. Pseudouridylate synthase, iii. Ribosomal proteins L13, S9, iv. Extradiol ring cleavage dioxygenase, v. phosphoglucosamine mutase.
(0.04 MB DOC)

Table S1 Genes that are predicted to be exported by the Tat system.
(0.04 MB DOC)

Table S2 Genes found in *H.orenii* without homologs in other Firmicutes.
(0.21 MB DOC)

Table S3 Genes with homologs within Firmicutes with significantly better hits in phyla outside Firmicutes.
(0.34 MB DOC)
