# Supplementary material for: Genome Analysis of the Anaerobic Thermohalophilic Bacterium Halothermothrix orenii
Source: PLoS One. 2009 Jan 15;4(1):e4192. doi: 10.1371/journal.pone.0004192 (PMC2626281; doi:10.1371/journal.pone.0004192)
Supplement: Figure S1 — The ribosomal operons in H.orenii. Red: 16S RNA, Blue: 23S RNA, Green: 5S RNA. (0.03 MB DOC) [file pone.0004192.s001.doc]

## Supplementary Material


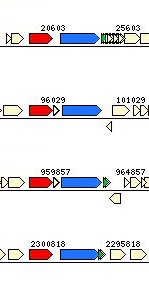


**Figure S1.** The ribosomal operons in *H.orenii*. Red: 16S RNA, Blue: 23S RNA, Green: 5S RNA.
