# Supplementary material for: Genome Analysis of the Anaerobic Thermohalophilic Bacterium Halothermothrix orenii
Source: PLoS One. 2009 Jan 15;4(1):e4192. doi: 10.1371/journal.pone.0004192 (PMC2626281; doi:10.1371/journal.pone.0004192)
Supplement: Figure S2 — Gene order around genes belonging to the lipidA pathway. Genes for H.orenii have been marked. Homologous genes in other genomes are indicated with same colors. (0.04 MB DOC) [file pone.0004192.s002.doc]

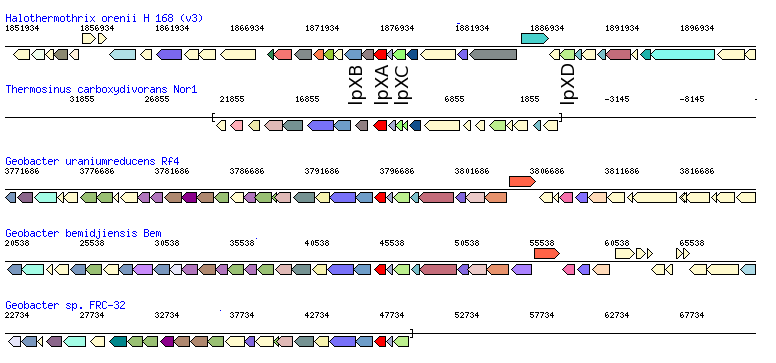


**Figure S2**. Gene order around genes belonging to the lipidA pathway. Genes for *H.orenii* have been marked. Homologous genes in other genomes are indicated with same colors.
