# Supplementary material for: Genome Analysis of the Anaerobic Thermohalophilic Bacterium Halothermothrix orenii
Source: PLoS One. 2009 Jan 15;4(1):e4192. doi: 10.1371/journal.pone.0004192 (PMC2626281; doi:10.1371/journal.pone.0004192)
Supplement: Figure S3 — Neighbor-joining tree of concatenated sequences of genes belonging to the lipidA pathway. (0.23 MB DOC) [file pone.0004192.s003.doc]

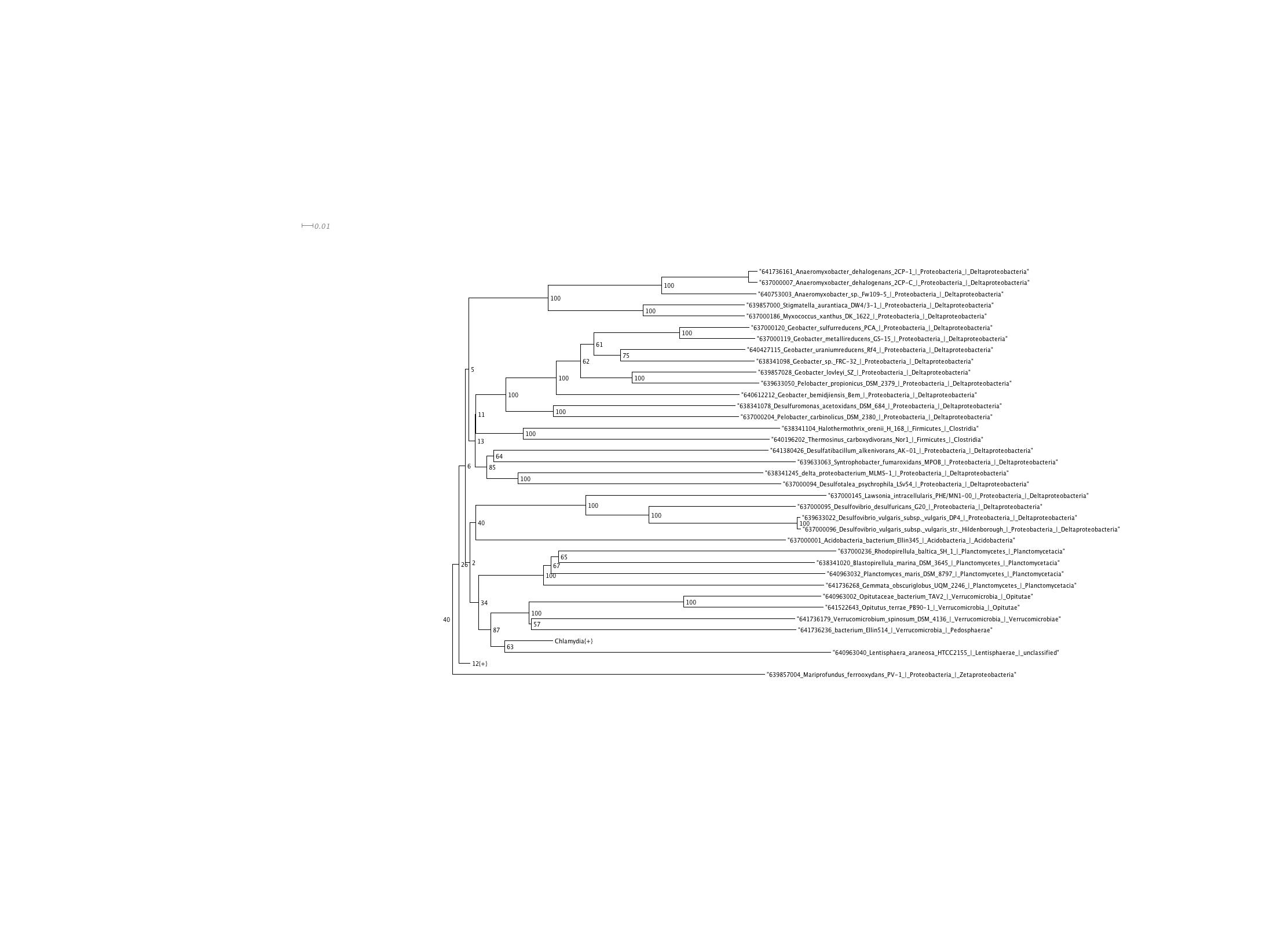


Figure S3. Neighbor-joining tree of concatenated sequences of genes belonging to the lipidA pathway.
