# Supplementary material for: Genome Analysis of the Anaerobic Thermohalophilic Bacterium Halothermothrix orenii
Source: PLoS One. 2009 Jan 15;4(1):e4192. doi: 10.1371/journal.pone.0004192 (PMC2626281; doi:10.1371/journal.pone.0004192)
Supplement: Figure S4 — Residue frequency in Mesophilic, Thermophilic, and halophilic bacteria compared to residue frequency in H.orenii. White boxes indicate residues with significant difference in their abundance between the different classes of organisms. (0.15 MB DOC) [file pone.0004192.s004.doc]

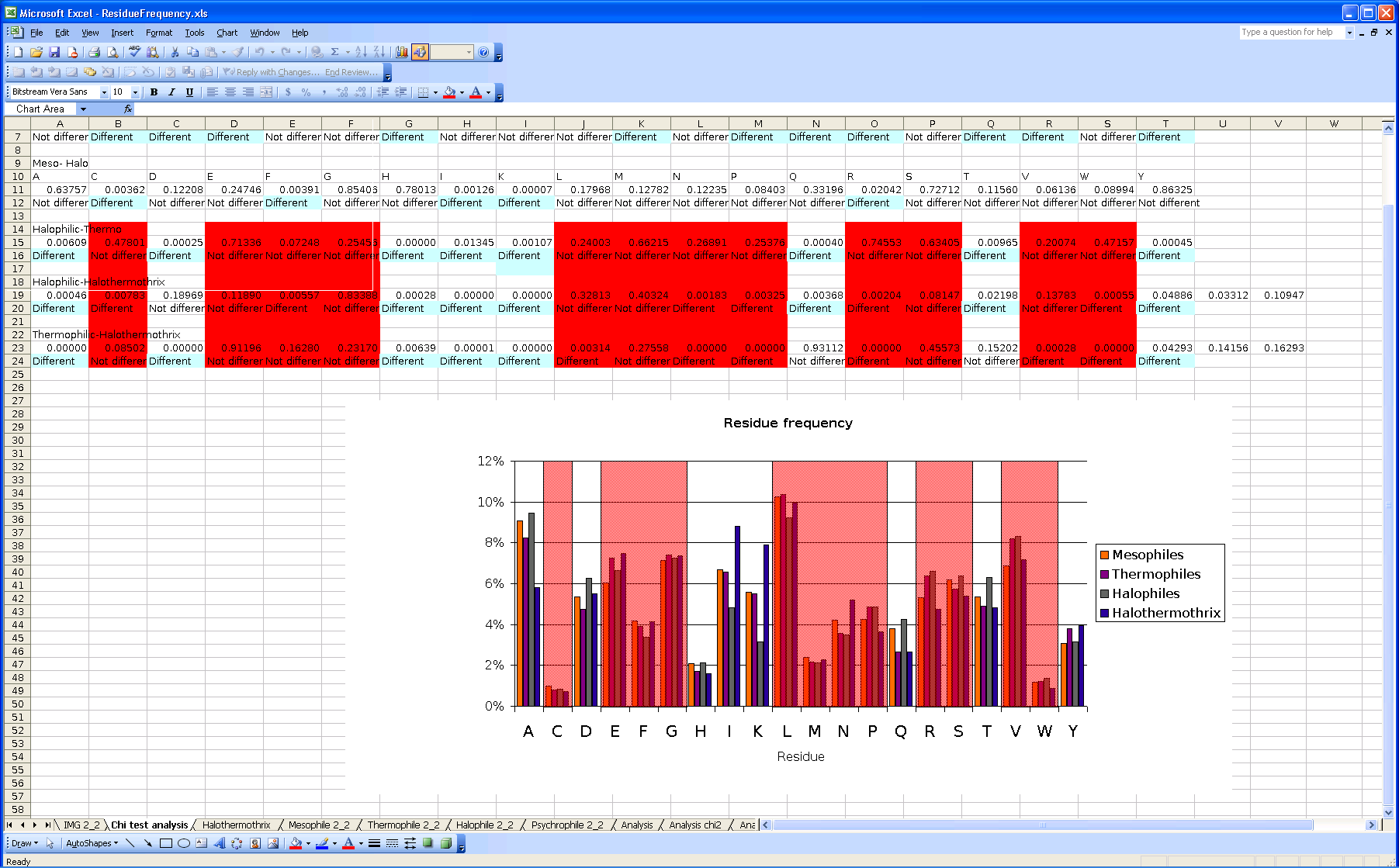


**Figure S4.** Residue frequency in Mesophilic, Thermophilic, and halophilic bacteria compared to residue frequency in *H.orenii.* White boxes indicate residues with significant difference in their abundance between the different classes of organisms.
