# Supplementary material for: Genome Analysis of the Anaerobic Thermohalophilic Bacterium Halothermothrix orenii
Source: PLoS One. 2009 Jan 15;4(1):e4192. doi: 10.1371/journal.pone.0004192 (PMC2626281; doi:10.1371/journal.pone.0004192)
Supplement: Figure S5 — Recent duplication in H.orenii genome. i. cobalt transporter, ii. Pseudouridylate synthase, iii. Ribosomal proteins L13, S9, iv. Extradiol ring cleavage dioxygenase, v. phosphoglucosamine mutase. (0.04 MB DOC) [file pone.0004192.s005.doc]

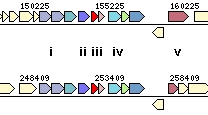


**Figure S5.** Recent duplication. i. cobalt transporter, ii. Pseudouridylate synthase, iii. Ribosomal proteins L13, S9, iv. Extradiol ring cleavage dioxygenase, v. phosphoglucosamine mutase.
