# Supplementary material for: Genome Analysis of the Anaerobic Thermohalophilic Bacterium Halothermothrix orenii
Source: PLoS One. 2009 Jan 15;4(1):e4192. doi: 10.1371/journal.pone.0004192 (PMC2626281; doi:10.1371/journal.pone.0004192)
Supplement: Table S1 — Genes that are predicted to be exported by the Tat system. (0.04 MB DOC) [file pone.0004192.s006.doc]

| N terminus of protein | Product name | Locus tag |
| --- | --- | --- |
| LFRRLRNLFLTGVLVLLPLIASV | Uncharacterized conserved protein, COG2928 | Hore_12550 |
| LNILNKMKTNYKYRRKLMGFLFIIPSMIVFLFFIFFPIVDSFRLS | binding-protein-dependent transport systems inner membrane component | Hore_20620 |
| LSIFFIKKFFRKSRRIGVIPRIIRNENA | histone family protein DNA-binding protein | Hore_10570 |
| LTSYIIRRLLILPIILLGVTLLIFSMIMCLSPYQRVA | binding-protein-dependent transport systems inner membrane component | Hore_04910 |
| MEIFGPIPSRRLGKSLGINIIPPKVCS | Radical SAM domain protein | Hore_04120 |
| MELSGAITEKRREKKMKRSFKYLVPIIISVFLLSLPVAAEV | hypothetical protein | Hore_23210 |
| MELSRRTIKRGIILSVSVSIVALILVMVLTQNELTFKSFS | conserved hypothetical protein | Hore_17180 |
| MIKIAWRNILRNKRRSLLILGIIIFGVMVLFLVKG | ABC-type transport system, involved in lipoprotein release, permease component | Hore_19660 |
| MKRRTALTLMVAVSLLLIITVSIQA | hypothetical protein | Hore_07120 |
| MKVFRRRLFIVLFSLLLLISVITSARA | alpha amylase | Hore_18730 |
| MLANLHKRRLLVFLVISLVVFTGLNLYTGSEAIA | alpha amylase | Hore_23200 |
| MLLFRRRVVILTLFFITIISVYL | hypothetical protein | Hore_12340 |
| MPDRRIVKELRNLEPGNLSTRTLSYLALFVAFTAVA | Predicted membrane protein | Hore_21280 |
| MPSQFYRRVIVLMVLFLSLLVLTLTGCEQN | SpoIID/LytB domain | Hore_21150 |
| MRFRRSVFLFLALVLVISFSVLA | peptidil-prolyl cis-trans isomerase | Hore_21140 |
| MRRLISKPEGHAGIFFIVVFVVMSLVSIRASA | hypothetical protein | Hore_16510 |
| MRRLTVLFVIIVMITTVFITGNASPACT | Uncharacterized protein conserved in bacteria, COG4254 | Hore_18170 |
| MRRRGYFLETLIFLLVLLVVTQVFLPGLAEKKIKEA | hypothetical protein | Hore_16420 |
| MVKNTMIIKEFRRLFILFIILIIIITGFSSYIVQA | Organic solvent tolerance protein OstA-like | Hore_17430 |
| MVKRRFILVLAVLLLGIVLLAGCGDKQEEG | Substrate-binding region of ABC-type glycine betaine transport system | Hore_18420 |
| MVVMRRYTPFSVAIIILLLFIMGSGSVYS | beta-glucosidase | Hore_19810 |
| VRRNIIFFLVLIFVLGVGSIALA | NLPA lipoprotein | Hore_22280 |

**Table S1**. Genes that are predicted to be exported by the Tat system.
