# Supplementary material for: Genome Analysis of the Anaerobic Thermohalophilic Bacterium Halothermothrix orenii
Source: PLoS One. 2009 Jan 15;4(1):e4192. doi: 10.1371/journal.pone.0004192 (PMC2626281; doi:10.1371/journal.pone.0004192)
Supplement: Table S2 — Genes found in H.orenii without homologs in other Firmicutes. (0.21 MB DOC) [file pone.0004192.s007.doc]

| Locus Tag | Product Name | Protein size |
| --- | --- | --- |
| Hore_00200 | hypothetical protein | 195 |
| Hore_00230 | hypothetical protein | 93 |
| Hore_00310 | Predicted nucleic acid-binding protein, contains PIN domain | 134 |
| Hore_00320 | hypothetical protein | 82 |
| Hore_00470 | hypothetical protein | 38 |
| Hore_00530 | Uncharacterized conserved protein, COG1507 | 179 |
| Hore_01720 | hypothetical protein | 51 |
| Hore_02170 | hypothetical protein | 44 |
| Hore_02280 | Uncharacterized conserved protein, COG1833 | 138 |
| Hore_02310 | hypothetical protein | 121 |
| Hore_02360 | hypothetical protein | 239 |
| Hore_02680 | hypothetical protein | 38 |
| Hore_03030 | hypothetical protein | 93 |
| Hore_03390 | hypothetical protein | 199 |
| Hore_03450 | MYND finger. | 289 |
| Hore_03480 | hypothetical protein | 172 |
| Hore_03530 | hypothetical protein | 73 |
| Hore_03540 | hypothetical protein | 95 |
| Hore_03550 | hypothetical protein | 115 |
| Hore_03560 | hypothetical protein | 241 |
| Hore_03570 | hypothetical protein | 145 |
| Hore_03580 | hypothetical protein | 69 |
| Hore_03590 | hypothetical protein | 59 |
| Hore_03600 | hypothetical protein | 56 |
| Hore_03610 | hypothetical protein | 106 |
| Hore_03650 | hypothetical protein | 43 |
| Hore_04220 | hypothetical protein | 624 |
| Hore_04300 | hypothetical protein | 35 |
| Hore_04350 | hypothetical protein | 228 |
| Hore_04360 | hypothetical protein | 219 |
| Hore_04390 | hypothetical protein | 113 |
| Hore_04540 | hypothetical protein | 146 |
| Hore_04750 | Dinitrogenase iron-molybdenum cofactor biosynthesis protein | 130 |
| Hore_04950 | hypothetical protein | 241 |
| Hore_04960 | hypothetical protein | 226 |
| Hore_05320 | hypothetical protein | |
| Hore_05350 | hypothetical protein | 96 |
| Hore_05540 | hypothetical protein | 86 |
| Hore_05570 | hypothetical protein | 58 |
| Hore_05730 | hypothetical protein | 125 |
| Hore_05790 | hypothetical protein | 495 |
| Hore_05850 | hypothetical protein | 76 |
| Hore_06080 | hypothetical protein | 202 |
| Hore_06420 | hypothetical protein | 90 |
| Hore_06430 | Peptidoglycan-binding domain 1 protein | 547 |
| Hore_06700 | hypothetical protein | 63 |
| Hore_07030 | hypothetical protein | 41 |
| Hore_07120 | hypothetical protein | 161 |
| Hore_07260 | hypothetical protein | 378 |
| Hore_07390 | hypothetical protein | 187 |
| Hore_07530 | hypothetical protein | 164 |
| Hore_07690 | hypothetical protein | 64 |
| Hore_08320 | hypothetical protein | 178 |
| Hore_08340 | hypothetical protein | 217 |
| Hore_08370 | hypothetical protein | 152 |
| Hore_08460 | hypothetical protein | 262 |
| Hore_08470 | hypothetical protein | 387 |
| Hore_08710 | hypothetical protein | 30 |
| Hore_08940 | hypothetical protein | 69 |
| Hore_09400 | hypothetical protein | 120 |
| Hore_09660 | alpha-2-macroglobulin domain protein | 1823 |
| Hore_09920 | hypothetical protein | 221 |
| Hore_10500 | hypothetical protein | 149 |
| Hore_10590 | hypothetical protein | 134 |
| Hore_10600 | hypothetical protein | 219 |
| Hore_10640 | hypothetical protein | 100 |
| Hore_10700 | hypothetical protein | 56 |
| Hore_10960 | hypothetical protein | 114 |
| Hore_10990 | hypothetical protein | 67 |
| Hore_11090 | hypothetical protein | 178 |
| Hore_11110 | hypothetical protein | 120 |
| Hore_11250 | hypothetical protein | 248 |
| Hore_11370 | hypothetical protein | 56 |
| Hore_11380 | hypothetical protein | 93 |
| Hore_11390 | hypothetical protein | 83 |
| Hore_11400 | hypothetical protein | 100 |
| Hore_11410 | hypothetical protein | 79 |
| Hore_11420 | hypothetical protein | 90 |
| Hore_11430 | protein serine/threonine kinase | 119 |
| Hore_11730 | hypothetical protein | 54 |
| Hore_11820 | hypothetical protein | 57 |
| Hore_11950 | phosphatidylglycerophosphatase( EC:3.1.3.27 ) | 146 |
| Hore_12360 | hypothetical protein | 71 |
| Hore_12370 | hypothetical protein | 82 |
| Hore_12460 | hypothetical protein | 47 |
| Hore_12640 | hypothetical protein | 128 |
| Hore_12650 | hypothetical protein | 246 |
| Hore_12670 | hypothetical protein | 138 |
| Hore_12680 | hypothetical protein | 244 |
| Hore_12690 | hypothetical protein | 180 |
| Hore_13030 | hypothetical protein | 153 |
| Hore_13080 | lipoprotein, putative | 612 |
| Hore_13090 | lipoprotein, putative | 198 |
| Hore_13260 | hypothetical protein | 362 |
| Hore_13270 | hypothetical protein | 120 |
| Hore_13300 | hypothetical protein | 170 |
| Hore_13500 | hypothetical protein | 265 |
| Hore_13620 | hypothetical protein | 91 |
| Hore_13630 | hypothetical protein | |
| Hore_13660 | hypothetical protein | 147 |
| Hore_13680 | hypothetical protein | 344 |
| Hore_13690 | Ribbon-helix-helix protein, copG family. | 83 |
| Hore_13770 | hypothetical protein | 182 |
| Hore_13790 | hypothetical protein | 99 |
| Hore_13900 | hypothetical protein | 197 |
| Hore_13950 | hypothetical protein | 114 |
| Hore_13960 | hypothetical protein | 356 |
| Hore_14000 | Sugar phosphate isomerases/epimerases( EC:3.1.21.2 ) | 254 |
| Hore_14470 | hypothetical protein | 131 |
| Hore_14970 | hypothetical protein | |
| Hore_15010 | Thioredoxin-like protein | 84 |
| Hore_15670 | hypothetical protein | 262 |
| Hore_15780 | hypothetical protein | 51 |
| Hore_15910 | hypothetical protein | 263 |
| Hore_16030 | Membrane-anchored protein predicted to be involved in regulation of amylopullulanase-like | 313 |
| Hore_16360 | hypothetical protein | 109 |
| Hore_16500 | hypothetical protein | |
| Hore_16510 | hypothetical protein | 182 |
| Hore_16600 | hypothetical protein | 193 |
| Hore_16640 | hypothetical protein | 119 |
| Hore_17210 | hypothetical protein | 34 |
| Hore_17240 | Radical SAM domain protein | 542 |
| Hore_17320 | hypothetical protein | 406 |
| Hore_17330 | hypothetical protein | 507 |
| Hore_17340 | hypothetical protein | 357 |
| Hore_17360 | hypothetical protein | 163 |
| Hore_17390 | hypothetical protein | 288 |
| Hore_17400 | hypothetical protein | 173 |
| Hore_17420 | hypothetical protein | 229 |
| Hore_17450 | hypothetical protein | 382 |
| Hore_17520 | hypothetical protein | 198 |
| Hore_17690 | hypothetical protein | 129 |
| Hore_17850 | hypothetical protein | 36 |
| Hore_18040 | hypothetical protein | 198 |
| Hore_18090 | hypothetical protein | 133 |
| Hore_18250 | hypothetical protein | 311 |
| Hore_18450 | hypothetical protein | 161 |
| Hore_18460 | hypothetical protein | 92 |
| Hore_18540 | hypothetical protein | 179 |
| Hore_18560 | hypothetical protein | 84 |
| Hore_18570 | hypothetical protein | 313 |
| Hore_18580 | hypothetical protein | 221 |
| Hore_18590 | hypothetical protein | 179 |
| Hore_18600 | hypothetical protein | 72 |
| Hore_18630 | hypothetical protein | 195 |
| Hore_18640 | Fimbrial assembly family protein | 434 |
| Hore_18650 | prepilin-type N-terminal cleavage/methylation domain | 190 |
| Hore_18660 | prepilin-type N-terminal cleavage/methylation domain | 120 |
| Hore_18670 | prepilin-type N-terminal cleavage/methylation domain | 146 |
| Hore_18710 | hypothetical protein | 210 |
| Hore_18720 | Type II secretory pathway component PulK-like | 347 |
| Hore_19000 | hypothetical protein | 490 |
| Hore_19200 | Pyrimidine reductase, riboflavin biosynthesis | 321 |
| Hore_19220 | Methenyltetrahydromethanopterin cyclohydrolase( EC:3.5.4.27 ) | 323 |
| Hore_19270 | hypothetical protein | 675 |
| Hore_19600 | hypothetical protein | 233 |
| Hore_19610 | hypothetical protein | 132 |
| Hore_19630 | hypothetical protein | 317 |
| Hore_19640 | hypothetical protein | 350 |
| Hore_19650 | hypothetical protein | 250 |
| Hore_19820 | hypothetical protein | 86 |
| Hore_19920 | hypothetical protein | 360 |
| Hore_20000 | hypothetical protein | 32 |
| Hore_20140 | hypothetical protein | |
| Hore_20400 | hypothetical protein | 59 |
| Hore_20430 | hypothetical protein | 483 |
| Hore_20440 | hypothetical protein | 164 |
| Hore_20550 | hypothetical protein | 185 |
| Hore_20810 | hypothetical protein | 82 |
| Hore_20980 | hypothetical protein | 43 |
| Hore_21250 | hypothetical protein | 306 |
| Hore_21330 | hypothetical protein | 163 |
| Hore_21420 | hypothetical protein | 138 |
| Hore_21440 | hypothetical protein | 128 |
| Hore_21600 | hypothetical protein | 146 |
| Hore_21610 | hypothetical protein | |
| Hore_21730 | hypothetical protein | 94 |
| Hore_21740 | hypothetical protein | 77 |
| Hore_21980 | hypothetical protein | 667 |
| Hore_22010 | hypothetical protein | 119 |
| Hore_22210 | polysaccharide export protein | 295 |
| Hore_22260 | Hemerythrin HHE cation binding domain protein | 142 |
| Hore_22330 | hypothetical protein | 80 |
| Hore_22380 | hypothetical protein | 223 |
| Hore_22430 | hypothetical protein | 235 |
| Hore_22520 | hypothetical protein | 248 |
| Hore_22760 | hypothetical protein | 116 |
| Hore_22840 | sulfotransferase | 328 |
| Hore_23000 | hypothetical protein | 31 |
| Hore_23150 | hypothetical protein | 60 |
| Hore_23180 | hypothetical protein | 561 |
| Hore_23190 | alpha-glucosidase | 701 |
| Hore_23210 | hypothetical protein | 372 |
| Hore_23220 | putative sigma E regulatory protein, MucB/RseB | 248 |

**Table S2**. Genes found in *H.orenii* without homologs in other Firmicutes.
