# Supplementary material for: Genome Analysis of the Anaerobic Thermohalophilic Bacterium Halothermothrix orenii
Source: PLoS One. 2009 Jan 15;4(1):e4192. doi: 10.1371/journal.pone.0004192 (PMC2626281; doi:10.1371/journal.pone.0004192)
Supplement: Table S3 — Genes with homologs within Firmicutes with significantly better hits in phyla outside Firmicutes. (0.34 MB DOC) [file pone.0004192.s008.doc]

| Locus Tag | Product Name | protein size | origin |
| --- | --- | --- | --- |
| Hore_00260 | hypothetical protein | 290 | Proteobacteria, Chlorobi |
| Hore_00270 | nucleotide-binding protein containing TIR -like domain | 274 | Proteobacteria, Cyanobacteria |
| Hore_00310 | Predicted nucleic acid-binding protein, contains PIN domain | 134 | Chlorobi |
| Hore_00320 | hypothetical protein | 82 | Cyanobacteria, Chlorobi, Spirochaetes |
| Hore_00380 | extracellular solute-binding protein family 1 | 422 | Deinococcus-Thermus, Proteobacteria |
| Hore_00390 | binding-protein-dependent transport systems inner membrane component | 382 | Deinococcus-Thermus, Proteobacteria |
| Hore_00400 | binding-protein-dependent transport systems inner membrane component | 463 | Proteobacteria, Deinococcus-Thermus |
| Hore_00430 | beta-phosphoglucomutase( EC, 5.4.2.6 ) | 216 | Chloroflexi, Cyanobacteria |
| Hore_00530 | Uncharacterized conserved protein, COG1507 | 179 | Thermotogae |
| Hore_00780 | extracellular solute-binding protein family 1 | 411 | Actinobacteria, Proteobacteria |
| Hore_00940 | CBS domain containing protein | 262 | Thermotogae, Euryarchaeota, Proteobacteria, Crenarchaeota, Chloroflexi |
| Hore_01710 | putative DNA helicase | 754 | Euryarchaeota, Bacteroidetes, Thermotogae |
| Hore_01880 | DNA alkylation repair enzyme | 236 | Thermotogae, Bacteroidetes, Chlorobi |
| Hore_01940 | pyrroline-5-carboxylate reductase( EC, 1.5.1.2 ) | 266 | Thermotogae, Proteobacteria, Euryarchaeota, Planctomycetes |
| Hore_02280 | Uncharacterized conserved protein, COG1833 | 138 | Proteobacteria, Euryarchaeota, Cyanobacteria, Chloroflexi, Bacteroidetes |
| Hore_02370 | hypothetical protein | 283 | Bacteroidetes, Actinobacteria, Deinococcus-Thermus |
| Hore_02410 | alpha amylase( EC, 3.2.1.98 ) | 364 | Euryarchaeota, Bacteroidetes |
| Hore_02640 | Uncharacterized conserved protein, COG2320 | 158 | Euryarchaeota |
| Hore_02670 | glycoside hydrolase family 16 | 503 | Bacteroidetes, Proteobacteria |
| Hore_02860 | P-loop kinase or ATPase distantly related to phosphoenolpyruvate carboxykinase | 591 | Thermotogae |
| Hore_02960 | NADH dehydrogenase I subunit E( EC, 1.6.5.3 ) | 136 | Thermotogae |
| Hore_02970 | NADH dehydrogenase I subunit F( EC, 1.6.5.3 ) | 408 | Thermotogae |
| Hore_03300 | binding-protein-dependent transport systems inner membrane component | 308 | Proteobacteria, Actinobacteria |
| Hore_03310 | binding-protein-dependent transport systems inner membrane component | 288 | Proteobacteria, Actinobacteria |
| Hore_03340 | extracellular solute-binding protein family 1 | 439 | Proteobacteria, Actinobacteria |
| Hore_03430 | hypothetical protein | 153 | unclassified, Fungi |
| Hore_03470 | DNA primase (bacterial type) | 815 | Planctomycetes |
| Hore_03540 | hypothetical protein | 95 | Euryarchaeota, Alveolata, Bacteroidetes |
| Hore_03550 | hypothetical protein | 115 | Alveolata, Apicomplexa, Proteobacteria, Euryarchaeota, Chordata, Bacteroidetes, Fungi |
| Hore_03950 | aldo/keto reductase( EC, 1.1.1.- ) | 312 | Proteobacteria |
| Hore_04040 | None | 419 | Thermotogae |
| Hore_04070 | transcriptional regulator, GntR family | 234 | Thermotogae |
| Hore_04120 | Radical SAM domain protein | 311 | Euryarchaeota, Proteobacteria, Crenarchaeota |
| Hore_04130 | hypothetical protein | 864 | Fusobacteria |
| Hore_04140 | UvrD/REP helicase | 1036 | Fusobacteria |
| Hore_04180 | beta-N-acetylhexosaminidase( EC, 3.2.1.52 ) | 618 | Actinobacteria, Chloroflexi, Acidobacteria, Streptophyta, Proteobacteria |
| Hore_04210 | glycoside hydrolase family 16 | 281 | Thermotogae |
| Hore_04220 | hypothetical protein | 624 | Bacteroidetes, Cyanobacteria, unclassified, Proteobacteria, Actinobacteria, Apicomplexa |
| Hore_04240 | Glucan endo-1,3-beta-D-glucosidase( EC, 3.2.1.39 ) | 1290 | Thermotogae, Proteobacteria |
| Hore_04270 | extracellular solute-binding protein family 1 | 418 | Proteobacteria, Deinococcus-Thermus |
| Hore_04450 | restriction modification system DNA specificity domain | 422 | Euryarchaeota |
| Hore_04590 | binding-protein-dependent transport systems inner membrane component | 294 | Actinobacteria |
| Hore_04600 | binding-protein-dependent transport systems inner membrane component | 277 | Actinobacteria |
| Hore_04610 | extracellular solute-binding protein family 1 | 431 | Actinobacteria |
| Hore_04620 | ADP-ribosylation/Crystallin J1 | 329 | Actinobacteria |
| Hore_04630 | ADP-ribosylation/Crystallin J1 | 347 | Actinobacteria |
| Hore_04640 | ADP-ribosylation/Crystallin J1 | 443 | Actinobacteria |
| Hore_04650 | ADP-ribosylation/Crystallin J1 | 326 | Actinobacteria |
| Hore_04750 | Dinitrogenase iron-molybdenum cofactor biosynthesis protein | 130 | Euryarchaeota |
| Hore_04790 | OsmC family protein | 144 | Euryarchaeota, Bacteroidetes |
| Hore_04810 | major facilitator superfamily MFS_1 | 423 | Euryarchaeota, Crenarchaeota, Thermotogae, Spirochaetes, Chlorophyta |
| Hore_04900 | extracellular solute-binding protein family 5 | 601 | Thermotogae |
| Hore_04910 | binding-protein-dependent transport systems inner membrane component | 343 | Thermotogae |
| Hore_04920 | binding-protein-dependent transport systems inner membrane component | 292 | Thermotogae |
| Hore_04970 | Xanthine/uracil/vitamin C permease | 433 | Thermotogae |
| Hore_04990 | Deoxyribodipyrimidine photo-lyase type II (EC 4.1.99.3) (IMGterm, M) | 466 | Euryarchaeota, Proteobacteria, Actinobacteria, Bacteroidetes |
| Hore_05060 | FeoA family protein | 80 | Chloroflexi, Euryarchaeota |
| Hore_05070 | Ferrous iron transporter protein FeoB | 607 | Euryarchaeota, Chloroflexi |
| Hore_05110 | DNA methylase N-4/N-6 domain protein | 386 | Euryarchaeota, Proteobacteria |
| Hore_05300 | Rubrerythrin | 161 | Proteobacteria |
| Hore_05360 | Predicted exporters of the RND superfamily | 727 | Thermotogae |
| Hore_05850 | hypothetical protein | 76 | Proteobacteria, Crenarchaeota, Fungi |
| Hore_05920 | Ferrous iron transport protein FeoB | 480 | Proteobacteria, Cyanobacteria, Euryarchaeota |
| Hore_06370 | metallophosphoesterase | 464 | Euryarchaeota |
| Hore_06380 | hypothetical protein | 968 | Euryarchaeota |
| Hore_06400 | peptidase S8 and S53 subtilisin kexin sedolisin | 797 | Thermotogae |
| Hore_06430 | Peptidoglycan-binding domain 1 protein | 547 | Proteobacteria, Bacteroidetes, Chlorobi |
| Hore_07260 | hypothetical protein | 378 | Fusobacteria, Euryarchaeota, Chordata, Proteobacteria, Apicomplexa |
| Hore_07390 | hypothetical protein | 187 | Proteobacteria |
| Hore_07440 | PBS lyase HEAT domain protein repeat-containing protein | 381 | Cyanobacteria, Euryarchaeota, Proteobacteria, Chloroflexi |
| Hore_07480 | helix-turn-helix domain protein | 301 | Euryarchaeota, Proteobacteria, Chloroflexi |
| Hore_07530 | hypothetical protein | 164 | Proteobacteria |
| Hore_07690 | hypothetical protein | 64 | Proteobacteria, Fungi |
| Hore_07920 | helicase c2 | 822 | Proteobacteria, Fusobacteria, Chlamydiae |
| Hore_08180 | carboxylesterase( EC, 3.1.1.1 ) | 266 | Thermotogae, Spirochaetes |
| Hore_08230 | WD40 domain protein beta Propeller | 918 | Proteobacteria |
| Hore_08340 | hypothetical protein | 217 | Proteobacteria |
| Hore_08450 | efflux transporter, putative, hydrophobe/amphiphile efflux-3 (HAE3) family | 764 | Proteobacteria |
| Hore_08460 | hypothetical protein | 262 | Spirochaetes, Bacteroidetes, Proteobacteria |
| Hore_08470 | hypothetical protein | 387 | Proteobacteria |
| Hore_08500 | None | 163 | Cyanobacteria, Proteobacteria |
| Hore_08910 | Predicted permeases | 247 | Bacteroidetes, Thermotogae, Proteobacteria, Chlorobi |
| Hore_08920 | Carboxymuconolactone decarboxylase | 118 | Thermotogae, Proteobacteria, Bacteroidetes |
| Hore_09450 | major facilitator superfamily MFS_1 | 422 | Proteobacteria |
| Hore_09580 | L-fuculokinase( EC, 2.7.1.51 ) | 511 | Thermotogae, Proteobacteria |
| Hore_09620 | RDD domain containing protein | 302 | Proteobacteria |
| Hore_09640 | beta-lactamase domain protein | 298 | Thermotogae, Chloroflexi |
| Hore_09660 | alpha-2-macroglobulin domain protein | 1823 | Bacteroidetes |
| Hore_09670 | penicillin-binding protein 1C | 770 | Bacteroidetes, Proteobacteria |
| Hore_09700 | alpha amylase( EC, 3.2.1.1 ) | 426 | Thermotogae |
| Hore_09710 | PfkB domain protein | 305 | Actinobacteria |
| Hore_09770 | apolipoprotein N-acyltransferase( EC, 2.3.1.- ) | 488 | Proteobacteria, Spirochaetes |
| Hore_09920 | hypothetical protein | 221 | Actinobacteria |
| Hore_10640 | hypothetical protein | 100 | Thermotogae |
| Hore_10800 | TPR repeat-containing protein | 245 | Cyanobacteria, Fusobacteria |
| Hore_10890 | 3-isopropylmalate dehydrogenase (EC 1.1.1.85) (IMGterm, M) | 358 | Thermotogae, Euryarchaeota |
| Hore_10990 | hypothetical protein | 67 | Proteobacteria |
| Hore_11000 | beta-lactamase domain protein | 252 | Planctomycetes, Proteobacteria, Bacteroidetes, Acidobacteria, Chloroflexi, Spirochaetes |
| Hore_11060 | Radical SAM domain protein | 239 | Euryarchaeota |
| Hore_11110 | hypothetical protein | 120 | Proteobacteria, unclassified, Streptophyta, Fungi, Bacteroidetes, Chordata, Apicomplexa, Euryarchaeota, Cyanobacteria, Nanoarchaeota |
| Hore_11170 | glutaredoxin | 78 | Actinobacteria, Proteobacteria |
| Hore_11180 | nitroreductase | 176 | Euryarchaeota |
| Hore_11190 | Predicted enzyme of the cupin superfamily | 90 | Thermotogae, Cyanobacteria, Proteobacteria |
| Hore_11350 | PGAP1 family protein | 313 | Bacteroidetes, Cyanobacteria |
| Hore_11430 | protein serine/threonine kinase | 119 | Alveolata, unclassified, Chordata, Apicomplexa, Fungi |
| Hore_11620 | None | 494 | Thermotogae |
| Hore_11630 | TrkA-C domain protein | 207 | Thermotogae |
| Hore_11720 | Chloride channel core | 402 | Euryarchaeota, Actinobacteria, Proteobacteria, Chlorobi, Planctomycetes, Crenarchaeota, Acidobacteria |
| Hore_11770 | S-layer domain protein | 255 | Thermotogae, Deinococcus-Thermus |
| Hore_11950 | phosphatidylglycerophosphatase( EC, 3.1.3.27 ) | 146 | Proteobacteria, Chlorobi, Aquificae, Bacteroidetes, Acidobacteria |
| Hore_12660 | type II and III secretion system protein | 385 | Thermotogae |
| Hore_12680 | hypothetical protein | 244 | Thermotogae |
| Hore_12690 | hypothetical protein | 180 | Thermotogae |
| Hore_13040 | oligopeptide/dipeptide ABC transporter, ATPase subunit | 333 | Thermotogae, Euryarchaeota, Spirochaetes |
| Hore_13050 | oligopeptide/dipeptide ABC transporter, ATPase subunit | 322 | Thermotogae, Spirochaetes, Euryarchaeota |
| Hore_13060 | binding-protein-dependent transport systems inner membrane component | 485 | Thermotogae, Spirochaetes |
| Hore_13070 | binding-protein-dependent transport systems inner membrane component | 349 | Thermotogae, Spirochaetes |
| Hore_13080 | lipoprotein, putative | 612 | Thermotogae |
| Hore_13090 | lipoprotein, putative | 198 | Spirochaetes, Thermotogae |
| Hore_13180 | binding-protein-dependent transport systems inner membrane component | 284 | Cyanobacteria, Chloroflexi, Actinobacteria |
| Hore_13190 | binding-protein-dependent transport systems inner membrane component | 289 | Cyanobacteria, Proteobacteria, Actinobacteria, Chloroflexi |
| Hore_13200 | extracellular solute-binding protein family 1 | 424 | Cyanobacteria, Proteobacteria, Chloroflexi |
| Hore_13230 | Abortive infection protein | 326 | Actinobacteria |
| Hore_13300 | hypothetical protein | 170 | Cyanobacteria, Proteobacteria, Chlorobi, Apicomplexa |
| Hore_13440 | major facilitator superfamily MFS_1 | 425 | Chloroflexi |
| Hore_13450 | Isoprenylcysteine carboxyl methyltransferase | 212 | Euryarchaeota |
| Hore_13520 | Acetyltransferase( EC, 2.3.1.- ) | 185 | Proteobacteria |
| Hore_13540 | hypothetical protein | 265 | Euryarchaeota |
| Hore_13580 | Radical SAM domain protein | 446 | Chlorobi |
| Hore_13610 | hydrolase of the HAD superfamily-like | 161 | Euryarchaeota |
| Hore_13620 | hypothetical protein | 91 | Fungi, Bacteroidetes, Proteobacteria, Thermotogae |
| Hore_13630 | Multimeric flavodoxin WrbA | 123 | Chlorobi |
| Hore_13635 | Multimeric flavodoxin WrbA | 87 | Chlorobi |
| Hore_13637 | Multimeric flavodoxin WrbA | 191 | Chlorobi |
| Hore_13650 | NADPH-dependent FMN reductase | 70 | Chlorobi |
| Hore_13660 | hypothetical protein | 147 | Proteobacteria |
| Hore_13690 | Ribbon-helix-helix protein, copG family. | 83 | Spirochaetes |
| Hore_13700 | PIN domain protein | 140 | Spirochaetes |
| Hore_13750 | hypothetical protein | 242 | Euryarchaeota, Proteobacteria |
| Hore_13830 | None | 274 | Proteobacteria, Cyanobacteria |
| Hore_13860 | hypothetical protein | 286 | Actinobacteria |
| Hore_13900 | hypothetical protein | 197 | Bacteroidetes |
| Hore_13940 | amidohydrolase 2 | 244 | Proteobacteria |
| Hore_13950 | hypothetical protein | 114 | Apicomplexa, Proteobacteria, Bacteroidetes |
| Hore_13970 | NUDIX hydrolase | 146 | Bacteroidetes, Deinococcus-Thermus |
| Hore_14000 | Sugar phosphate isomerases/epimerases( EC, 3.1.21.2 ) | 254 | Proteobacteria, Bacteroidetes, Euryarchaeota, Spirochaetes |
| Hore_14530 | binding-protein-dependent transport systems inner membrane component | 284 | Cyanobacteria, Chloroflexi, Actinobacteria |
| Hore_14540 | binding-protein-dependent transport systems inner membrane component | 289 | Cyanobacteria, Chloroflexi, Proteobacteria |
| Hore_14550 | extracellular solute-binding protein family 1 | 432 | Cyanobacteria, Chloroflexi, Proteobacteria |
| Hore_14640 | SPFH domain, Band 7 family protein (IMGterm, M) | 326 | Proteobacteria, Bacteroidetes |
| Hore_14650 | Membrane protein implicated in regulation of membrane protease activity | 144 | Proteobacteria |
| Hore_14700 | transcriptional regulator, TetR family | 207 | Euryarchaeota |
| Hore_14730 | hypothetical protein | 274 | Proteobacteria, Bacteroidetes |
| Hore_14780 | 4-aminobutyrate aminotransferase( EC, 2.6.1.19 ) | 437 | Acidobacteria, Streptophyta |
| Hore_14960 | Ankyrin | 483 | Fungi, Cyanobacteria |
| Hore_14980 | glycosyl transferase family 2 | 239 | Proteobacteria, Chloroflexi, Bacteroidetes, Chlorobi, Actinobacteria |
| Hore_15010 | Thioredoxin-like protein | 84 | Proteobacteria, Fungi, Bacteroidetes, unclassified, Cyanobacteria, Streptophyta |
| Hore_15020 | binding-protein-dependent transport systems inner membrane component | 285 | Actinobacteria, Proteobacteria |
| Hore_15030 | binding-protein-dependent transport systems inner membrane component | 289 | Actinobacteria, Proteobacteria |
| Hore_15040 | extracellular solute-binding protein family 1 | 458 | Proteobacteria, Actinobacteria |
| Hore_15080 | hypothetical protein | 97 | Euryarchaeota |
| Hore_15130 | CRISPR-associated RAMP protein, Cmr4 family | 325 | Bacteroidetes, Euryarchaeota |
| Hore_15140 | CRISPR-associated protein, Cmr3 family | 393 | Bacteroidetes, Proteobacteria |
| Hore_15160 | CRISPR-associated RAMP protein, Cmr1 family | 288 | Bacteroidetes, Aquificae |
| Hore_15220 | CRISPR-associated helicase Cas3 | 822 | Euryarchaeota |
| Hore_15230 | CRISPR-associated protein Cas5 | 238 | Euryarchaeota |
| Hore_15250 | CRISPR-associated protein, Csh1 family | 691 | Euryarchaeota |
| Hore_15290 | heat shock protein DnaJ domain protein | 107 | Proteobacteria, Euryarchaeota, Arthropoda |
| Hore_15540 | binding-protein-dependent transport systems inner membrane component | 277 | Proteobacteria, Chloroflexi, Actinobacteria |
| Hore_15550 | binding-protein-dependent transport systems inner membrane component | 294 | Chloroflexi, Proteobacteria |
| Hore_15560 | extracellular solute-binding protein family 1 | 418 | Chloroflexi, Proteobacteria |
| Hore_15570 | glycoside hydrolase family 2 sugar binding | 744 | Proteobacteria |
| Hore_15920 | Squalene synthase( EC, 2.5.1.21 ) | 321 | Cyanobacteria, Euryarchaeota, Streptophyta |
| Hore_15940 | Extracellular ligand-binding receptor | 370 | Thermotogae, Euryarchaeota, Proteobacteria |
| Hore_15950 | nitroreductase | 188 | Thermotogae |
| Hore_16030 | Membrane-anchored protein predicted to be involved in regulation of amylopullulanase-like | 313 | Thermotogae, Euryarchaeota, Deinococcus-Thermus |
| Hore_16510 | hypothetical protein | 182 | Euryarchaeota, Bacteroidetes, Proteobacteria |
| Hore_16560 | Flagellar basal body P-ring biosynthesis protein-like | 336 | Proteobacteria, Thermotogae, Spirochaetes |
| Hore_17240 | Radical SAM domain protein | 542 | Euryarchaeota, Thermotogae, Crenarchaeota |
| Hore_17270 | type I phosphodiesterase/nucleotide pyrophosphatase | 547 | Chloroflexi, Acidobacteria |
| Hore_17290 | type I phosphodiesterase/nucleotide pyrophosphatase | 453 | Euryarchaeota |
| Hore_17320 | hypothetical protein | 406 | Proteobacteria |
| Hore_17330 | hypothetical protein | 507 | Cyanobacteria, Euryarchaeota, Proteobacteria, Fungi, Nematoda, Chlorobi |
| Hore_17340 | hypothetical protein | 357 | Proteobacteria |
| Hore_17570 | UDP-3-O-[3-hydroxymyristoyl] N-acetylglucosamine deacetylase( EC, 3.5.1.- ) | 288 | Proteobacteria, Spirochaetes, Chlamydiae, Cyanobacteria, Bacteroidetes, Fusobacteria, Acidobacteria, Chlorobi |
| Hore_17620 | peptidase S8 and S53 subtilisin kexin sedolisin | 595 | Thermotogae, Cyanobacteria |
| Hore_18100 | SufBD protein | 316 | Euryarchaeota, Chloroflexi, Thermotogae |
| Hore_18110 | ABC transporter related | 241 | Thermotogae, Euryarchaeota |
| Hore_18170 | Uncharacterized protein conserved in bacteria, COG4254 | 378 | Alveolata, Apicomplexa, Nematoda, Chordata, Proteobacteria, Fungi, Spirochaetes |
| Hore_18210 | Sucrose-phosphate synthase( EC, 2.4.1.14 ) | 496 | Thermotogae, Cyanobacteria |
| Hore_18240 | alpha amylase( EC, 3.2.1.98 ) | 623 | Thermotogae |
| Hore_18260 | glycoside hydrolase family 13 domain protein | 900 | Thermotogae, unclassified, Euryarchaeota |
| Hore_18420 | Substrate-binding region of ABC-type glycine betaine transport system | 316 | Cyanobacteria |
| Hore_18460 | hypothetical protein | 92 | Proteobacteria |
| Hore_18570 | hypothetical protein | 313 | Spirochaetes |
| Hore_18640 | Fimbrial assembly family protein | 434 | Proteobacteria, Crenarchaeota |
| Hore_18650 | prepilin-type N-terminal cleavage/methylation domain | 190 | Proteobacteria |
| Hore_18670 | prepilin-type N-terminal cleavage/methylation domain | 146 | Proteobacteria |
| Hore_18680 | type II secretion system protein G | 143 | Proteobacteria |
| Hore_18720 | Type II secretory pathway component PulK-like | 347 | Aquificae, Proteobacteria |
| Hore_18730 | alpha amylase( EC, 3.2.1.98 ) | 442 | Bacteroidetes, Euryarchaeota |
| Hore_18740 | polar amino acid ABC transporter, inner membrane subunit | 222 | Thermotogae |
| Hore_18760 | polar amino acid ABC transporter, inner membrane subunit | 216 | Thermotogae |
| Hore_18770 | extracellular solute-binding protein family 3 | 244 | Thermotogae |
| Hore_18810 | phosphoesterase PA-phosphatase related | 224 | Proteobacteria |
| Hore_18820 | glycosyl transferase family 39 | 465 | Aquificae, Proteobacteria |
| Hore_18840 | hypothetical protein | 308 | Bacteroidetes, Chlorobi |
| Hore_18860 | None | 367 | Proteobacteria, Chlorobi |
| Hore_18950 | Uncharacterized protein conserved in bacteria, COG2121 | 222 | Proteobacteria, Spirochaetes, Acidobacteria, Fusobacteria, Chlorobi |
| Hore_19200 | Pyrimidine reductase, riboflavin biosynthesis | 321 | Acidobacteria, Euryarchaeota, Actinobacteria, Chloroflexi |
| Hore_19220 | Methenyltetrahydromethanopterin cyclohydrolase( EC, 3.5.4.27 ) | 323 | Euryarchaeota, Proteobacteria |
| Hore_19270 | hypothetical protein | 675 | Bacteroidetes, Proteobacteria |
| Hore_19410 | V-type ATP synthase subunit E( EC, 3.6.3.15 ) | 202 | Euryarchaeota |
| Hore_19520 | xylose isomerase( EC, 5.3.1.5 ) | 354 | Actinobacteria |
| Hore_19600 | hypothetical protein | 233 | Alveolata, Apicomplexa, Fungi, Proteobacteria, Fusobacteria |
| Hore_19610 | hypothetical protein | 132 | Fusobacteria, Bacteroidetes, Proteobacteria, Crenarchaeota, Thermotogae, Cyanobacteria, unclassified, Apicomplexa |
| Hore_19630 | hypothetical protein | 317 | unclassified, Alveolata, Bacteroidetes, Proteobacteria, Cyanobacteria, Euryarchaeota, Planctomycetes |
| Hore_19640 | hypothetical protein | 350 | Chordata, Alveolata, Proteobacteria, Chlorobi |
| Hore_19650 | hypothetical protein | 250 | Proteobacteria, Chlorobi |
| Hore_19660 | ABC-type transport system, involved in lipoprotein release, permease component | 400 | Proteobacteria |
| Hore_19820 | hypothetical protein | 86 | Proteobacteria |
| Hore_19900 | extracellular solute-binding protein family 1 | 415 | Proteobacteria, Actinobacteria |
| Hore_19950 | nucleotide kinase-like | 180 | Thermotogae |
| Hore_20050 | hypothetical protein | 721 | Proteobacteria, Bacteroidetes |
| Hore_20080 | redox-active disulfide protein 2 | 77 | Euryarchaeota, Thermotogae, Proteobacteria, Chlorobi |
| Hore_20110 | major facilitator superfamily MFS_1 | 447 | Euryarchaeota |
| Hore_20130 | Na+/Ca+ antiporter, CaCA family | 356 | Spirochaetes |
| Hore_20210 | restriction modification system DNA specificity domain | 565 | Cyanobacteria, Proteobacteria |
| Hore_20420 | conserved repeat domain | 1248 | Proteobacteria |
| Hore_20430 | hypothetical protein | 483 | Thermotogae, Euryarchaeota |
| Hore_20450 | binding-protein-dependent transport systems inner membrane component | 277 | Proteobacteria, Actinobacteria |
| Hore_20460 | binding-protein-dependent transport systems inner membrane component | 321 | Proteobacteria, Actinobacteria |
| Hore_20470 | extracellular solute-binding protein family 1 | 406 | Proteobacteria |
| Hore_20500 | Monosaccharide-transporting ATPase( EC, 3.6.3.17 ) | 291 | Thermotogae, Proteobacteria, Actinobacteria |
| Hore_20510 | binding-protein-dependent transport systems inner membrane component | 290 | Thermotogae, Proteobacteria |
| Hore_20520 | extracellular solute-binding protein family 1 | 417 | Proteobacteria |
| Hore_20540 | regulatory protein LuxR | 887 | Chloroflexi |
| Hore_20580 | glycoside hydrolase family 43 | 315 | Bacteroidetes |
| Hore_20600 | glycoside hydrolase family 1 | 424 | Actinobacteria |
| Hore_20810 | hypothetical protein | 82 | Spirochaetes |
| Hore_20870 | extracellular solute-binding protein family 1 | 430 | Proteobacteria, Deinococcus-Thermus |
| Hore_21440 | hypothetical protein | 128 | Arthropoda, Proteobacteria, Cyanobacteria |
| Hore_21560 | phosphate ABC transporter, inner membrane subunit PstA | 284 | Euryarchaeota, Proteobacteria, Bacteroidetes |
| Hore_21610 | phosphate ABC transporter, permease protein PstC | 200 | Proteobacteria, Euryarchaeota, Bacteroidetes |
| Hore_21620 | phosphate binding protein | 273 | Proteobacteria, Euryarchaeota, Chlorobi |
| Hore_21980 | hypothetical protein | 667 | Proteobacteria |
| Hore_22170 | adenylosuccinate lyase( EC, 4.3.2.2 ) | 462 | Spirochaetes |
| Hore_22210 | polysaccharide export protein | 295 | Proteobacteria, Thermotogae |
| Hore_22220 | lipopolysaccharide biosynthesis protein | 474 | Thermotogae, Cyanobacteria |
| Hore_22260 | Hemerythrin HHE cation binding domain protein | 142 | Proteobacteria, Crenarchaeota, Chordata, Apicomplexa |
| Hore_22310 | Auxin Efflux Carrier | 318 | Thermotogae |
| Hore_22330 | hypothetical protein | 80 | Thermotogae |
| Hore_22350 | Uncharacterized conserved protein, COG1434 | 256 | Thermotogae, Aquificae |
| Hore_22380 | hypothetical protein | 223 | Actinobacteria, Cyanobacteria |
| Hore_22400 | Patatin | 343 | Actinobacteria, Proteobacteria, Bacteroidetes, Chloroflexi |
| Hore_22440 | Glycosyl hydrolase family 32 domain protein | 210 | Proteobacteria, Actinobacteria, Planctomycetes, Bacteroidetes |
| Hore_22510 | hypothetical protein | 199 | Bacteroidetes, Fungi, Alveolata, Apicomplexa, Nematoda |
| Hore_22520 | hypothetical protein | 248 | Chloroflexi |
| Hore_22580 | CheD, stimulates methylation of MCP proteins | 168 | Proteobacteria, Planctomycetes |
| Hore_22610 | CheA signal transduction histidine kinase | 699 | Proteobacteria |
| Hore_22710 | ABC-type sugar transport system, periplasmic component( EC, 3.6.3.17 ) | 308 | Proteobacteria |
| Hore_22800 | glycosyl transferase group 1 | 359 | Chlorobi, Proteobacteria, Cyanobacteria |
| Hore_22840 | sulfotransferase | 328 | Proteobacteria, Cyanobacteria |
| Hore_22850 | polysaccharide biosynthesis protein | 499 | Euryarchaeota |
| Hore_22900 | lipopolysaccharide biosynthesis protein | 278 | Euryarchaeota |
| Hore_22970 | alpha-mannosidase( EC, 3.2.1.24 ) | 1054 | Chlorobi |
| Hore_22980 | binding-protein-dependent transport systems inner membrane component | 273 | Thermotogae |
| Hore_22990 | binding-protein-dependent transport systems inner membrane component | 286 | Thermotogae |
| Hore_23010 | extracellular solute-binding protein family 1 | 436 | Thermotogae |
| Hore_23190 | alpha-glucosidase | 701 | Bacteroidetes, Proteobacteria, Actinobacteria |
| Hore_23220 | putative sigma E regulatory protein, MucB/RseB | 248 | Proteobacteria |
| Hore_23230 | ABC-type transport system, involved in lipoprotein release, permease component | 419 | Proteobacteria, Euryarchaeota |
| Hore_23240 | ABC-type transport system, involved in lipoprotein release, permease component | 415 | Proteobacteria, Bacteroidetes, Euryarchaeota |
| Hore_23260 | ABC-type transport system, involved in lipoprotein release, permease component | 419 | Proteobacteria |
| Hore_23270 | ABC-type transport system, involved in lipoprotein release, permease component | 411 | Proteobacteria, Euryarchaeota, Bacteroidetes |
| Hore_23400 | TPR repeat-containing protein | 183 | Cyanobacteria, Acidobacteria, Bacteroidetes, Proteobacteria |

**Supplementary table 3.** Genes with homologs within Firmicutes with significantly better hits in phyla outside Firmicutes.
